# Identification of Immune Infiltration-Related ceRNAs as Novel Biomarkers for Prognosis of Patients With Primary Open-Angle Glaucoma

**DOI:** 10.3389/fgene.2022.838220

**Published:** 2022-05-27

**Authors:** Daowei Zhang, Jiawen Wu, Shenghai Zhang, Jihong Wu

**Affiliations:** ^1^ Eye and ENT Hospital, College of Medicine, Eye Institute, Fudan University, Shanghai, China; ^2^ Shanghai Key Laboratory of Visual Impairment and Restoration, Science and Technology Commission of Shanghai Municipality, Shanghai, China; ^3^ State Key Laboratory of Medical Neurobiology, Institutes of Brain Science and Collaborative Innovation Center for Brain Science, Shanghai, China; ^4^ Key Laboratory of Myopia, Ministry of Health, Shanghai, China

**Keywords:** glaucoma, POAG, ceRNA network, trabecular meshwork, enrichment analysis

## Abstract

Glaucoma is the leading cause of irreversible blindness globally; hence, relevant clinical biomarkers are necessary to enable diagnosis, early detection, and development of novel therapies. The differentially expressed genes were annotated and visualized using Gene Ontology and Kyoto Encyclopedia. In addition, a competitive endogenous ribonucleic acids network was constructed using Cytoscape, which explained the regulation of gene expression in glaucoma. The CIBERSORT algorithm was employed to analyze the immune microenvironment. We validated that the core genes could predict glaucoma occurrence and development and identified potential molecular mechanism pathways, which were associated with immune infiltration and participated in endogenous regulation networks. Our data may partially explain the pathogenesis of glaucoma and they provide potential theoretical support for targeted therapy.

## Introduction

Glaucoma, the leading cause of irreversible blindness globally, is commonly regarded as a neurodegenerative disease with a multifactorial origin, resulting in progressive visual field loss and blindness (Beykin et al., 2021). It is estimated that by 2040, the number of patients with glaucoma will have increased from the prevailing 60 million to 111.8 million (Tham et al., 2014). Primary open-angle glaucoma (POAG) is the most prevalent glaucoma subtype. POAG combined with irreversible optic neuropathy keep increasing the global burden of glaucoma because of unsatisfactory clinical diagnosis and treatment thus far (Zimprich et al., 2020). Currently, the accurate diagnosis and detection of POAG at an early stage is difficult. Without efficient instruments to help estimate the screening and diagnosis, progression monitoring, and response to treatment, all diagnoses and detections are based on the clinician’s experience. Therefore, glaucoma-related biomarkers must be identified for clinical testing to enable early diagnosis and detection of disease progression. This will assist in clinical examinations, enhance early diagnosis, and improve early phase clinical trial design, allowing for timely intervention. Therefore, the emergence of gene therapy studies, regulation of gene expression, and gene networks aiming at different pathways involved in glaucoma pathogenesis are high-priority landmarks for ophthalmic research. Recent studies have demonstrated that long non-coding ribonucleic acids (lncRNAs), which do not encode proteins and are >200-nt-long RNAs, can mediate the pathogenesis of glaucoma (Yao et al., 2020) (Kong et al., 2021). According to existing reports, lncRNAs regulate gene expression, protein modification, cell differentiation, immune response, and other critical pathological processes (Jiang et al., 2019). Furthermore, some of these functions have been identified in neurodegenerative diseases such as Alzheimer’s disease (Zhang and Wang, 2021). The sponge hypothesis was proposed to suggest that lncRNAs suppressed miRNA interactions with mRNAs, thereby affecting the translation of protein-coding genes (Dhamija and Menon, 2018). Therefore, lncRNAs are called competing endogenous RNAs (ceRNAs), and the lncRNA–microRNA (miRNA)–messenger RNA (mRNA) regulation network is called the ceRNA network. In this study, we established the ceRNA network of core genes in glaucoma to describe the interactions of these RNAs.

The microenvironment of glaucoma comprises immune cells, extracellular matrix, various growth factors, and inflammatory factors and possesses special physical and chemical characteristics, which significantly affect the diagnosis and clinical treatment sensitivity (Gajewski et al., 2013). The resident immune cells in the retina are implicated in the pathogenesis of various neurodegenerative diseases, including multiple sclerosis and glaucoma, which affects the optical system (Namekata et al., 2019). A study showed that neurodegeneration in glaucoma was associated with immune cell infiltration in the optic nerve head region (Maddineni et al., 2020). Another study primarily tested the function of a complex immune infiltration called complement-mediated inflammation in glaucoma (Harder et al., 2020). The ocular hypertension model showed infiltrating monocytes in pathological glaucoma (Tribble et al., 2020). However, the exact role of immune infiltration in glaucoma is yet to be discovered.

This study used next-generation sequencing data to analyze individual trabecular meshwork (TM) samples from POAG patients. We matched controls to identify RNA expression characteristics and evaluated the potential of these RNAs to serve as biomarkers for POAG diagnosis and prognosis to explore further the underlying molecular mechanisms of core genes affecting glaucoma progression.

## Materials and Methods

### Data

**FIGURE 1:**
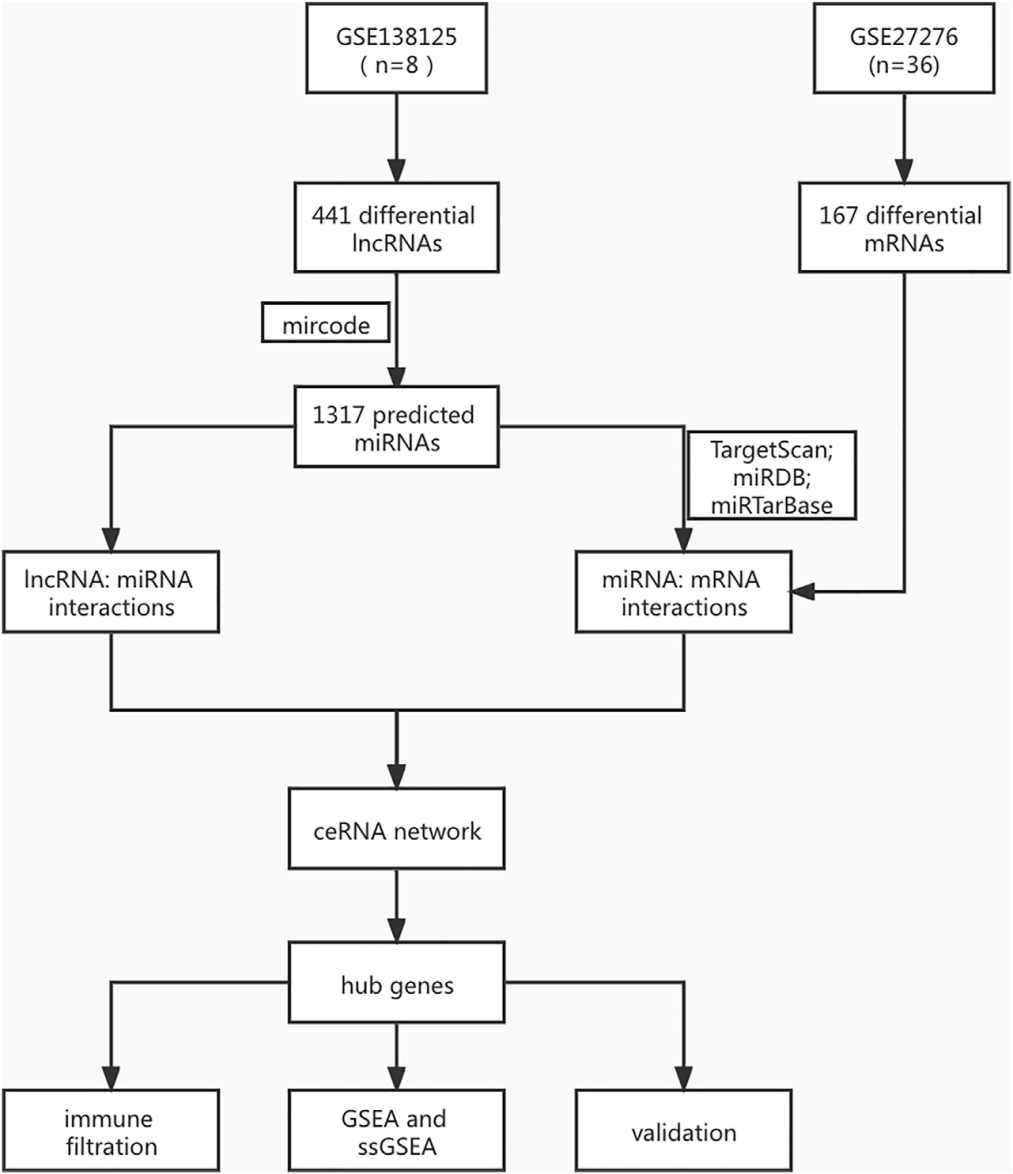
The identification process of POAG biomarkers.

The Series Matrix File data files of lncRNA were downloaded from the National Center for Biotechnology Information Gene Expression Omnibus (GEO); GSE138125 comprises the data of four normal samples (*n* = 4) and four POAG samples (*n* = 4). GSE27276 comprises the data of nineeen normal samples (*n* = 19) and seventeen POAG samples (*n* = 17). The POAG biomarkers identification process is shown in Figure 1. The limma package in R (Version 1.3.959) was applied to analyze the difference between the two datasets using the standard log2FC > 1 with adj.P.Val <0.05. In addition, we reserved differently expressed genes to explore the molecular mechanisms of POAG occurrence and development.

### Gene Ontology (GO) and Kyoto Encyclopedia of Genes and Genomes (KEGG)

We used GO and KEGG to annotate specific genes in order to identify the biological function and signaling pathways related to the occurrence and development of glaucoma. We defined significant results as Min overlap ≥3 and *p* ≤ 0.01.

### The lncRNA–miRNA–mRNA Network

The miRcode database was used to predict the interaction groups of lncRNA–miRNA. miRDB, mirtarbase, and TargetScan were combined to predict the interaction between miRNA and mRNA, and the co-identified targeted mRNAs were selected for further analysis. In addition, by evaluating the interactions between lncRNA–miRNA and mRNA–miRNA, Protein–protein interaction (PPI) network of RNAs was constructed and visualized using Cytoscape.

### The Correlation of Immune Genes

The RNA-seq data of different POAG patients’ subtypes were processed using the CIBERSORT algorithm (Chen et al., 2018) to estimate the relative infiltration proportions of 22 immune cells. The corrplot R package was used to analyze the relationship of immune cells and assess their interaction’s influence. The vioplot R package was applied to plot the relative number of immune cells and evaluate the influence of differently expressed genes on immune infiltration. Spearman correlation analysis was performed on gene expression level and immune cell numbers, with *p* < 0.05 considered significant.

### Gene Set Enrichment Analysis (GSEA) and Single Sample Gene Set Enrichment Analysis (ssGSEA)

Predefined gene sets were assessed using GSEA. Genes were ranked according to the degree of differential expression in lncRNA and mRNA samples, and the predefined gene sets were tested whether they were enriched at the top or bottom of the ranked list. In this study, we used GSEA to compare the difference in signaling pathways between the high- and low-risk groups, exploring a possible molecular mechanism of the difference in the prognosis of patients in the two groups. The substitution frequency was set as 1,000, and the type was set as phenotype. As ssGSEA classifying gene sets with common biological functions, chromosomal localization, and physiological regulation, we obtained immune cells and conducted Spearman correlation analysis with hub genes.

### Statistical Analyses

All statistical analyses were conducted in R language (version 3.6). All statistical tests were bilateral, and *p* < 0.05 indicated a significant difference.

## Results

### Differential Gene Expression and Enrichment

**FIGURE 2:**
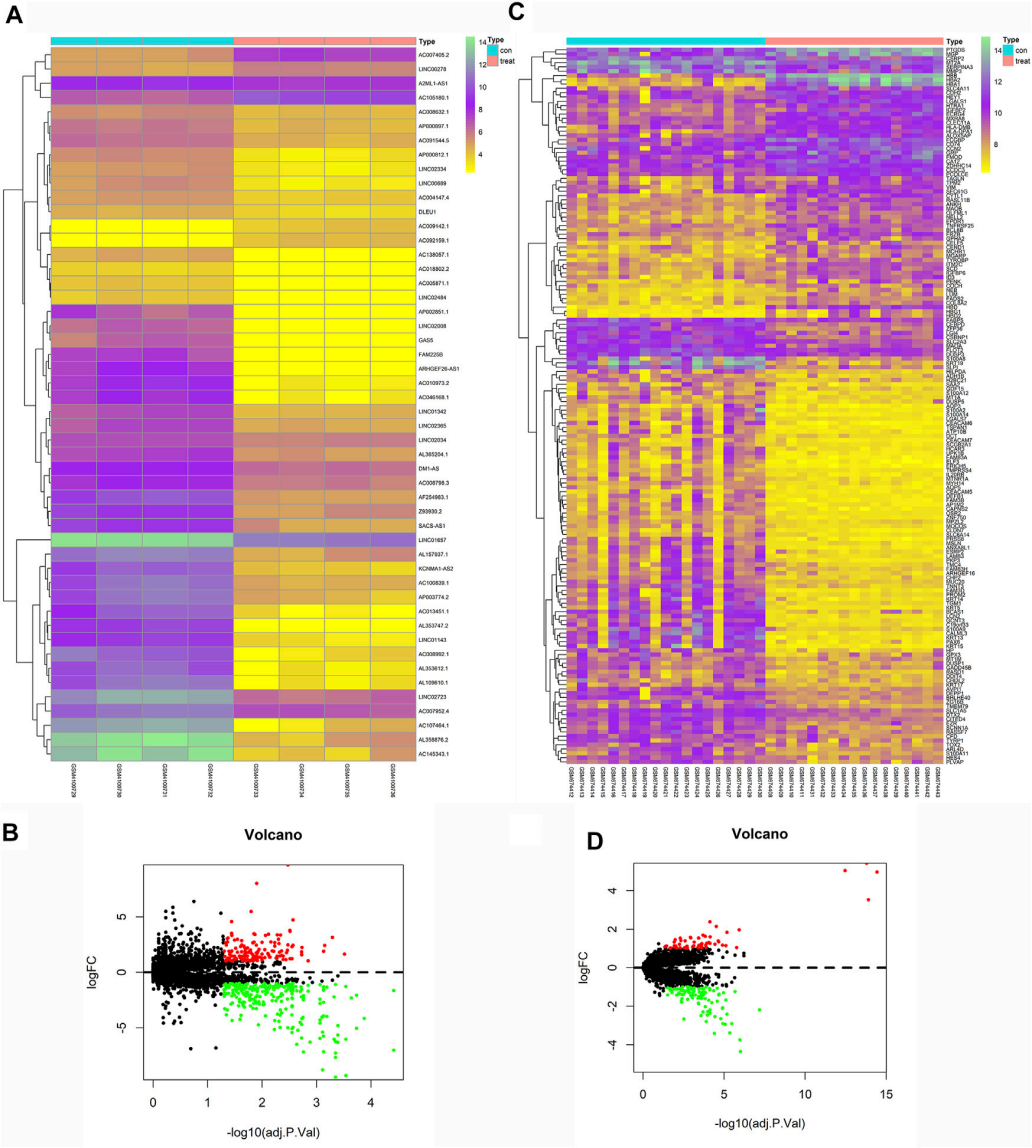
Heatmaps and Volcano Plots of GSE138125 and GSE27276. GSE138125 was a lncRNA transcriptome dataset. We screened a total of 411 differential genes, including 143 up-regulated genes and 268 down-regulated genes **(A,B)**. We also downloaded mRNA transcriptome data from GSE27276, there was 59 up-regulated genes and 108 down-regulated genes in all the 167 differential genes **(C,D)**.

**FIGURE 3:**
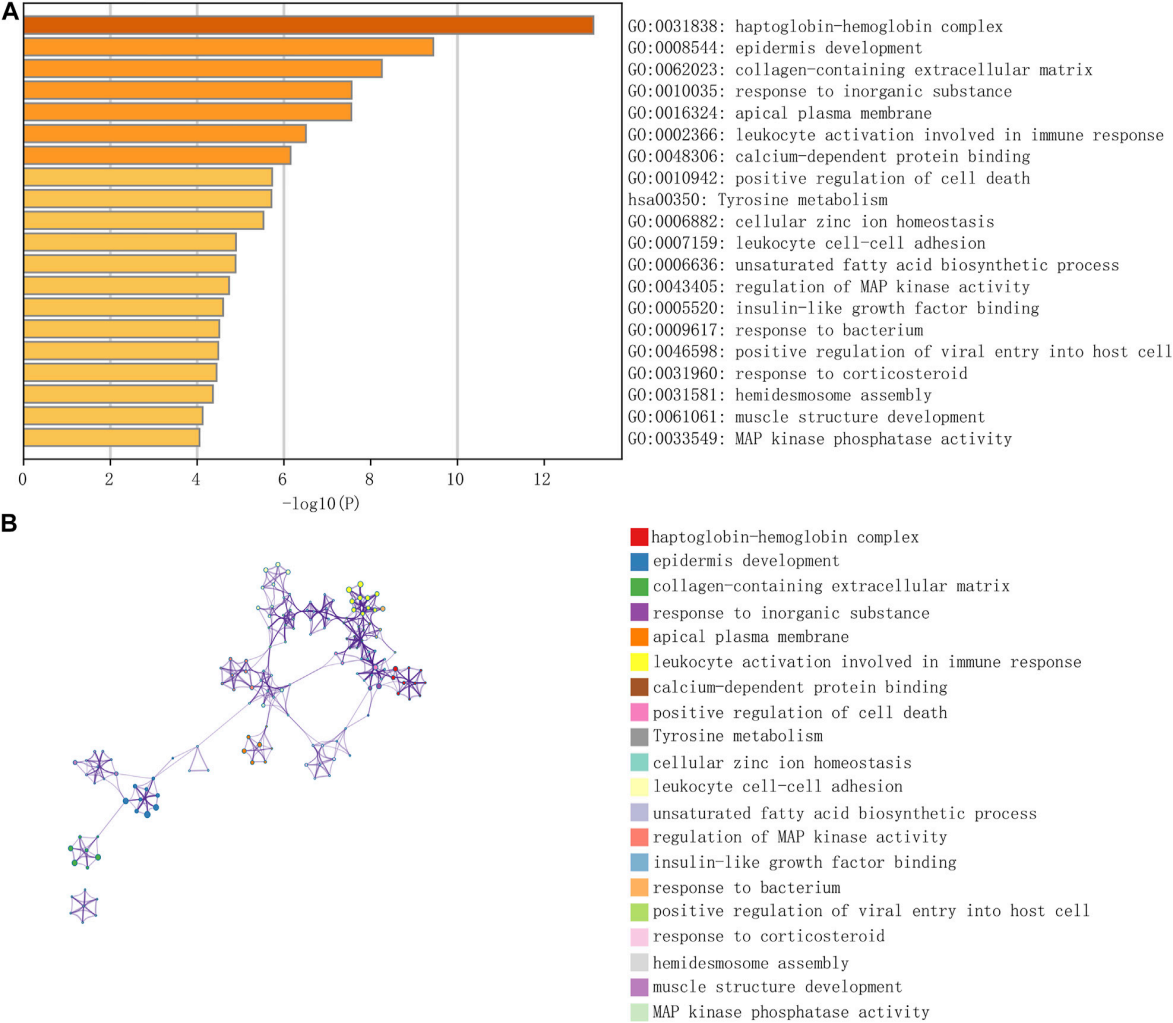
The enrichment of total 167 differential genes from GSE27276. The results of pathway analysis showed that differential genes were mainly enriched in 20 pathways, especially in haptoglobin hemoglobin complex and epidermis development **(A,B)**.

We downloaded eight groups of lncRNA transcriptome data of GSE138125 from GEO, comprising normal (*n* = 4) and POAG (*n* = 4) groups. We acquired 36 groups of RNA transcriptome data from GSE27276, comprising normal (*n* = 19) and POAG (*n* = 17) groups. First, the limma package was applied to screen the differentially expressed genes between normal and POAG groups with the following conditions: adj.P.Val <0.05 and | Log2FC | > 1. A total of 411 gene expression variations was identified from GSE138125, comprising 143 upregulated genes and 268 downregulated genes (Figures 2A,B). In addition, there were 59 upregulated genes and 108 downregulated genes in the 167 differentially expressed genes of GSE27276 (Figures 2C,D). Subsequently, pathway analysis was performed on these 167 differentially expressed genes, with the results showing that they were mainly enriched in 20 pathways, especially the haptoglobin–hemoglobin complex and epidermis development pathway (Figure 3).

### lncRNA–miRNA–mRNA Relationship

We used miRcode database (http://www.mircode.org/) to predict the miRNA targets of 411 different lncRNAs, and the results showed that there were 1,317 lncRNA-related targeted miRNAs, providing 1,317 pairs of lncRNA–miRNA. We used TargetScan, miRDB, and mirtarbase databases to predict miRNA-related target mRNAs, and 1,935 pairs of miRNA–mRNA were obtained. Subsequently, all the mRNAs from 1,935 miRNA–mRNA relationship pairs were intersected with 167 differentially expressed mRNAs, reserving eight pairs of miRNA–mRNA. Then, the eight pairs of miRNA–mRNA and 1,317 pairs of lncRNA–miRNA were intersected, and finally, 64 pairs of lncRNA–miRNA–mRNA were obtained.

### PPI Network

**FIGURE 4:**
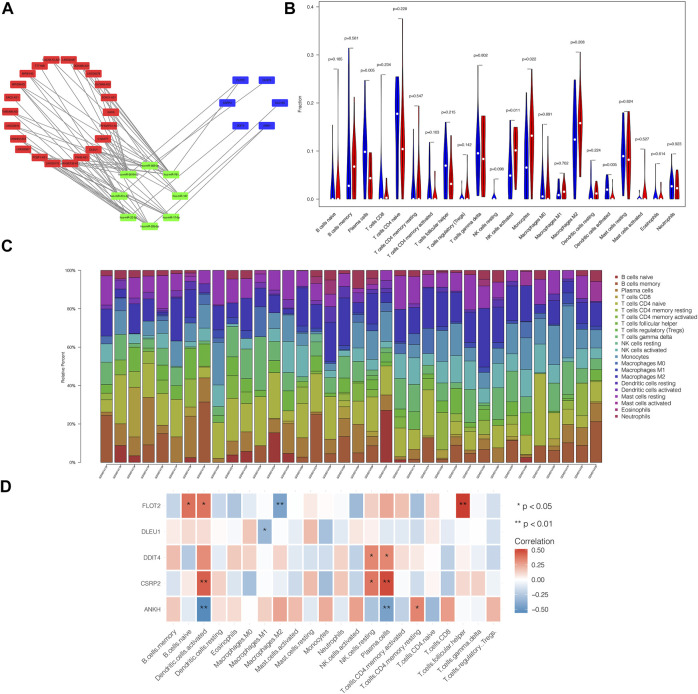
ceRNA network of differential genes and the relationship between core genes and immune infiltration. PPI network of 64 pairs of lncRNA-miRNA-mRNA relationship was generated, and then visualized by Cytoscape **(A)** and mRNAs with top five degree were defined as core genes, including FLOT2, ANKH, DLEU1, DDIT4 and CSRP2. Violin plot demonstrated that Plasma cells and Dendritic cells activated were significantly reduced in patients comparing with control group, while NK cells activated and Monocytes were increased remarkably **(B)**. **(C)** showed the relative percentage of 22 type of immune cells in each sample. We found that core genes were all closed related to the content of immune cells as we prospected **(D)**. In brief, ANKH was positively correlated with T cells CD4 memory resting and was negatively correlated with Dendritic cells activated and Plasma cells. CSRP2 was positively associated with Dendritic cells activated, NK cells resting and Plasma cells. DDIT was positively associated with NK cells resting and Plasma cells. There was a negative correlation between DLEU1 and Macrophages M1. FLOT2 had a positive relation with B cells naïve, Dendritic cells activated and T cells follicular helper, while having a negative relation with Macrophages M2.

PPI network of 64 pairs of lncRNA–miRNA–mRNA was generated and visualized using Cytoscape to analyze the competitive endogenous network of key genes in lncRNA and miRNA. Figure 4A shows the competitive endogenous network diagram of ceRNA. We calculated the connectivity degree of each gene with hub genes, defined as mRNAs with top five grades, including *FLOT2*, *ANKH*, *DLEU1*, *DDIT4*, and *CSRP2*.

### Association Between Core Genes and Immune Infiltration

By analyzing the relationship between hub genes and immune infiltration in POAG, the potential molecular mechanism of hub genes influencing glaucoma progression was further explored. The violin plot demonstrated that plasma cell and activated dendritic cell (DC) numbers were significantly reduced in patients compared to those in the control group. By contrast, natural killer cells (NK) were activated, and monocyte counts were increased remarkably (Figure 4B). Figure 4C shows the relative percentage of 22 types of immune cells in each sample. We observed that core genes were closely related to the immune cell counts as expected (Figure 4D). In brief, *ANKH* expression was positively correlated with memory resting CD4 T cell count and was negatively correlated with activated DC and plasma cell counts. *CSRP2* expression was positively associated with activated DC, resting NK cell, and plasma cell counts. *DDIT* expression was positively associated with resting NK cell and plasma cell counts. There was a negative correlation between *DLEU1* expression and M1 macrophage counts. *FLOT2* expression had a positive relation with naïve B cell, activated DC, and T follicular helper cell counts, whereas it was negatively correlated with M2 macrophage counts.

### Verification of Hub Genes

**FIGURE 5:**
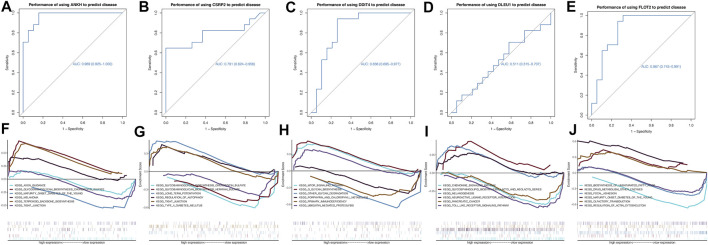
ROC curves and GSEA analyses of hub genes. The AUCs of each gene were ANKH-AUC: 0.969 (0.925–1.000), CSRP2-AUC: 0.791 (0.624–0.958), DDIT4-AUC: 0.836 (0.695–0.977), DLEU1-AUC: 0.511 (0.315–0.707) and FLOT2-AUC: 0.867 (0.743–0.991) individually **(A–E)**. GSEA analysis showed that ANKH enriched in AXON GUIDANCE, GLYCOSAMINOGLYCAN BIOSYNTHESIS CHONDROITIN SULFATE and MATURITY ONSET DIABETES OF THE YOUNG. CSRP2 enriched in GLYCOSAMINOGLYCAN BIOSYNTHESIS CHONDROITIN SULFATE and GLYCOSAMINOGLYCAN BIOSYNTHESIS HEPARAN SULFATE. DDIT4 enriched in MTOR SIGNALING PATHWAY and O GLYCAN BIOSYNTHESIS. DLEU1 enriched in CHEMOKINE SIGNALING PATHWAY and GLYCOSPHINGOLIPID BIOSYNTHESIS LACTO AND NEOLACTO SERIES. FLOT2 enriched in BIOSYNTHESIS OF UNSATURATED FATTY ACIDS and DRUG METABOLISM OTHER ENZYMES **(F–J)**.

**FIGURE 6:**
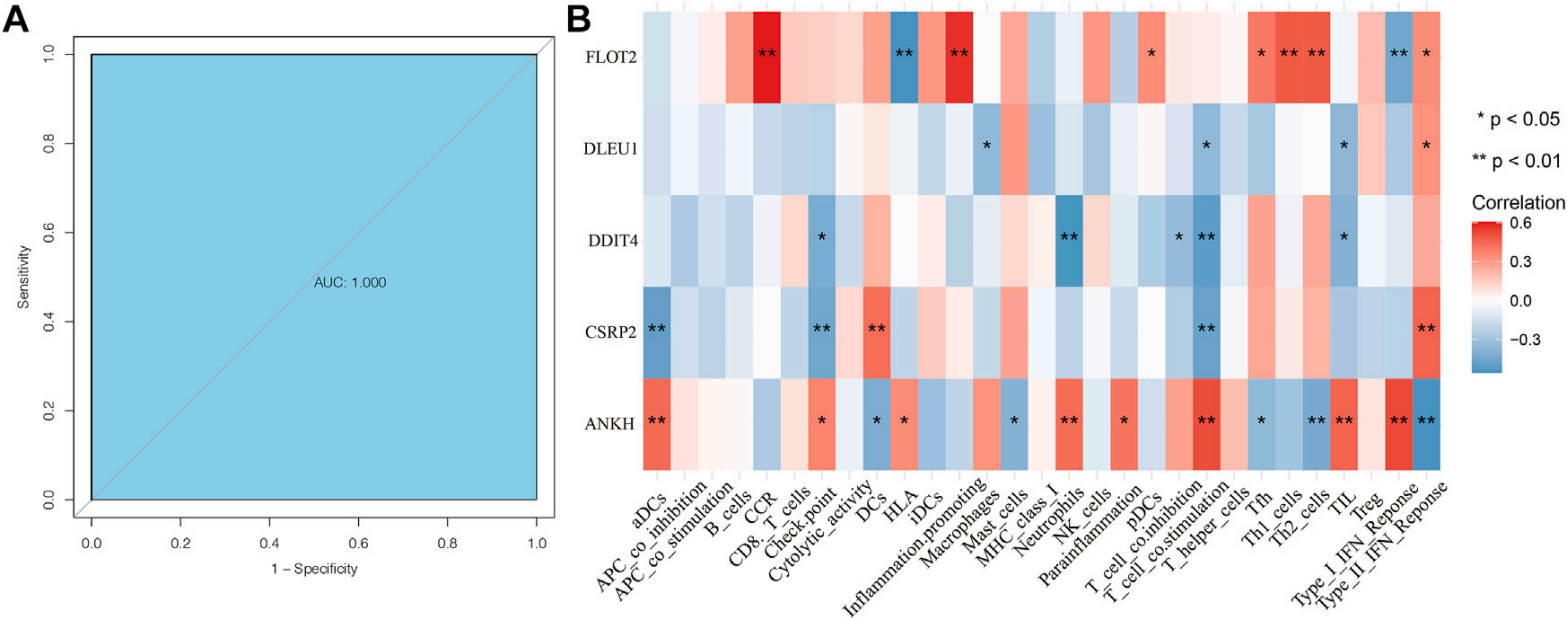
ROC curve of combined clinical biomarker and ssGSEA analyses of hub genes. The AUC of the combined clinical biomarker reached to 1 **(A)**. Hub genes were all related to immune cells **(B)**. FLOT2 was closely related to CCR, HLA, pDCs, Tfh, Th1_cells, Th2_cells, Type_I_IFN_Reponce and Type_II_IFN_Reponce. DLEU1 was related to Macrophages, T_cell_co.stimulation, TIL and Type_II_IFN_Reponce. DDIT4 was relative with Check. point, Netrophils, T_cell_co.inhibition, T_cell_co.stimulation and TIL all negatively. CSRP2 was closely related to aDCs, Check. point, DCs, T_cell_co.stimulation and Type_II_IFN_Reponce. ANKH was closely related to aDCs, Check. point, DCS, HLA, Mast_cells, Netrophils, Parainflammation, T_cell_co.stimulation, Tfh, Th2_cells,TIL, Type_I_IFN_Reponce and Type_II_IFN_Reponce.

According to the receiver operating characteristic curve used for diagnostic efficacy verification, the higher the area under the curve (AUC) value, the better the predictive efficiency. The results showed that the AUC values of *ANKH*, *CSRP2*, *DDIT4*, *DLEU1*, and *FLOT2* hub genes were in 0.969 (0.925–1.000), 0.791 (0.624–0.958), 0.836 (0.695–0.977), 0.511 (0.315–0.707), and 0.867 (0.743–0.991), respectively (Figures 5A–E), while the AUC values of these combined-five genes approached to 1 (Figure 6A). The results suggest that the *ANKH*, *CSRP2*, *DDIT4* and *FLOT2* can efficiently predict POAG occurrence and development separately. Moreover, the five hub genes impacted more effectively as a combined clinical biomarker.

### GSEA for Potential Molecular Mechanisms of Core Genes

We investigated specific signaling pathways related to hub genes and explored the potential molecular mechanism of POAG progression. The enrichment results are shown in Figures 5F–J. GSEA showed that *ANKH* was enriched in axon guidance, glycosaminoglycan biosynthesis chondroitin sulfate, and maturity-onset diabetes of the young. *CSRP2* was enriched in glycosaminoglycan, chondroitin sulfate, glycosaminoglycan, and heparan sulfate biosynthesis. *DDIT4* was enriched in mammalian target of rapamycin signaling pathway and O-glycan biosynthesis. *DLEU1* was enriched in the chemokine signaling pathway, glycosphingolipid biosynthesis and lacto and neolacto series. *FLOT2* was enriched in the biosynthesis of unsaturated fatty acids, drug metabolism, and other enzymes.

### ssGSEA for Hub Genes Related to Immune Cells

We performed ssGSEA analysis with Spearman correlation analysis between five core genes and ssGSEA immune cells. The results showed hub genes were all related to immune cells (Figure 6B). FLOT2 was closely related to CCR, HLA, pDCs, Tfh, Th1_cells, Th2_cells, Type_I_IFN_Reponce and Type_II_IFN_Reponce. DLEU1 was related to Macrophages, T_cell_co.stimulation, TIL and Type_II_IFN_Reponce. DDIT4 was relative with Check. point, Netrophils, T_cell_co.inhibition, T_cell_co.stimulation and TIL all negatively. CSRP2 was closely related to aDCs, Check. point, DCs, T_cell_co.stimulation and Type_II_IFN_Reponce. ANKH was closely related to aDCs, Check. point, DCS, HLA, Mast_cells, Netrophils, Parainflammation, T_cell_co.stimulation, Tfh, Th2_cells,TIL, Type_I_IFN_Reponce and Type_II_IFN_Reponce.

## Discussion

In this study, we constructed a ceRNA network in TM from patients with POAG and identified five core genes as potential biomarkers by screening and validating next-generation sequencing datasets. In recent years, ceRNA has attracted a lot of attention from academic groups. It is represented as a new view of the regulated gene expression module. ceRNA regulation is more sophisticated and complicated than miRNA regulation, which involves more RNA molecules, such as mRNA, pseudogenes of the coding gene, long non-coding RNAs, miRNA, and lncRNA. The miRcode (http://www.mircode.org/) is a public database used to query the relationship between lncRNAs and miRNAs. It covers the complete GENCODE-annotated transcriptome, including non-coding RNA genes over 10,000 in length. By intersecting the identified lncRNA–miRNA and miRNA–mRNA pairs, we constructed the ceRNA network. After calculating the degree of each gene, mRNAs with top five degrees were identified as core genes. In addition, the analysis of core genes and immune infiltration showed a strong correlation with immune cell counts. In addition, the five core genes showed a significant difference between patients and controls with regards to AUC, suggesting that they could predict POAG occurrence and development. We further explored the underlying molecular mechanisms of glaucoma progression, and the result showed 12 pathways were enriched, mainly related to immunity and metabolism. Moreover, ssGSEA showed hub genes were all highly correlated with immune cells. Thus, we assume that the core genes have potential clinical utility as POAG diagnostic markers based on immune infiltration.

Generally, TM in the juxtacanalicular region was mediated to reduce outflow resistance in the conventional outflow pathway and help maintain intraocular pressure (iop) homeostasis (Weinreb et al., 2020). However, induration and reconstruction of TM are involved in the elevated iop often associated with glaucoma. *ANKH* is the human homolog of the murine *ANK* gene (Kirsch et al., 2009). ANKH is a multichannel transmembrane protein that transports intracellular pyrophosphate (PPi) to the extracellular milieu, an amino acid comprising 492 base pairs (Mitton-Fitzgerald et al., 2016). It has been demonstrated that the loss of ANKH transporter function in mice led to excessive mineralization because of reduced PPi transport (Ho et al., 2000). Other studies indicated that *ANKH* took part in chondrocalcinosis articularis (Kumar et al., 2019), fibroblast ossification (He and Dong, 2020), and vascular smooth muscle cell calcification (Chen et al., 2019a), suggesting that *ANKH* may play a crucial role in the hardening and remodeling of TM cells in POAG patients. This view has been established in studies on POAG identifying novel risk loci (Choquet et al., 2018).

It is universally accepted that the flotillin family is correlated with vesicular invaginations of the plasma membrane and regulation of signal transduction (Zhao et al., 2011). *FLOT2* is an oncogene involved in the pathogenic process of several cancers (Liu et al., 2015) (Li et al., 2019). Some researchers believe that *FLOT2* is involved in various biological processes, including neuronal differentiation (Bodrikov et al., 2017), adhesion, endocytosis, embryo survival, and phagocytosis, and several signal pathways (Langhorst et al., 2005). In addition, a study on gestational diabetes mellitus showed that the phosphatidylinositol-3-kinase and protein kinase B pathway could be repressed by regulating *FLOT2* expression, suggesting *FLOT2* inhibition could alleviate insulin resistance and liver gluconeogenesis (Chen et al., 2019b). Thus, *FLOT2,* which is associated with the prognosis of a variety of diseases, may fail to serve as an independent POAG biomarker, although it has the potential to be an indicator for disease screening, progression, recurrence, and treatment efficacy.

Many factors are necessary for the development of glaucoma, such as aging, exogenous environmental factors, and endogenous factors, and one of the genetic factors that may determine glaucoma pathogenesis is response to hypoxia and nitric oxide (Lin et al., 2014). A recent study demonstrated that glaucoma progression could be accelerated by the interaction between hypoxia-inducible factor (HIF)-1α and vascular endothelial growth factor (Zhou et al., 2021a). *CSRP2* encodes the LIM domain protein CSRP2, which participates in regulating organ development and cell differentiation (Weiskirchen et al., 1997), and is widely expressed in various tissues (Jain et al., 1996). Although the precise cellular function of CSRP2 needs further intensive study, it is known that it can be upregulated in hypoxia via HIF-1-mediated transactivation of the CSRP2 promoter (Hoffmann et al., 2018). Thus, *CSRP2* may provide prognostic significance and potential therapeutic targets in POAG management.

DDIT4 is a highly conserved protein composed of 232 amino acids encoded by *DDIT4.* Although the expression of *DDIT4* is ubiquitously at low levels in most adult tissues (Shoshani et al., 2002), it can be induced by multiple cellular stresses, such as hypoxia (Brugarolas et al., 2004). Other scholars suggested that *DDIT4* was strongly connected to metabolic disorder changes and metabolic functions (Lipina and Hundal, 2016), including its potential contribution to mitochondrial biology. Another study revealed that it might be a potential biomarker for POAG (Zhou et al., 2021b). Therefore, it will be worthwhile to reassess the position of metabolism in glaucoma since more than one gene informs us about metabolic processes, such as the mammalian target of the rapamycin signaling pathway and the role O-glycan biosynthesis, play in this disease (Liu and Prokosch, 2021).

lncRNA *DLEU1* is a new research focus in cancer biology. It is regarded as a promising target for biotherapy in numerous tumors because it participates in various biological processes and indispensable role in the pathophysiology of diseases (Song et al., 2020). Furthermore, *DLEU1* has been incorporated in the immune prognostic signature in other diseases (Chen et al., 2020), emphasizing its relationship with immune infiltration and the microenvironment. *DLEU1* can also be a biomarker of non-cancerous diseases, like ischemic stroke (Fan et al., 2019), due to its involvement in regulating multiple biological processes and pathways. In addition, it offers an option for estimating the progression and therapeutic effect of the disease.

However, our study has some limitations. First, our results need further validation. Research on cellular and animal levels is required to explore how these potential biomarkers work in POAG progression. Second, since there are not many datasets of trabecular meshwork for glaucoma, we were limited by the source of samples. The size of our research was relatively small. In addition, difficulties in obtaining patients’ clinical information made it difficult to combine the study with clinical features.

## Conclusion

This study screened, provided, and validated five core genes as clinical biomarkers and potential therapeutic targets for POAG. In addition, this work comprehensively explained POAG biomarkers from an immunological perspective, making them more widely applicable in clinical practice. These results, however, need to be accepted and applied with caution.

## Data Availability

Publicly available datasets were analyzed in this study. This data can be found here: GSE138125 GSE27276.
